# Effect of a Seeding System on Competitive Performance of Elite Players During Major Tennis Tournaments

**DOI:** 10.3389/fpsyg.2020.01294

**Published:** 2020-06-26

**Authors:** Yixiong Cui, Yue Zhao, Haoyang Liu, Miguel-Ángel Gómez, Ran Wei, Yuanlong Liu

**Affiliations:** ^1^AI Sports Engineering Lab, School of Sports Engineering, Beijing Sport University, Beijing, China; ^2^School of Physical Education, Beijing Sport University, Beijing, China; ^3^Facultad de Ciencias de la Actividad Física y del Deporte–INEF, Universidad Politécnica de Madrid, Madrid, Spain; ^4^College of Education and Human Development, Western Michigan University, Kalamazoo, MI, United States; ^5^Department of Human Performance and Health Education, Western Michigan University, Kalamazoo, MI, United States

**Keywords:** professional tennis, key performance indicator, seeded players, discriminant analysis, match demands

## Abstract

The performance of professional tennis players in the four major Grand Slam tournaments has always been an important research topic, which advances the understanding of the current development of tennis. However, there is little known about the difference between higher-ranked and lower-ranked players considering match performance statistics. The study was aimed to explore the technical, tactical, and physical performance indicators that best discriminate seeded and non-seeded male players in Grand Slams. A total of 549 matches played by 189 individual players during 2015–2017 Grand Slam men’s singles were sampled, with corresponding match statistics gathered for each player observation, concerning players’ serving, returning, net point, break point, efficiency, and physical performance. The results showed that the seeded players outperformed the non-seeded players in serve and return, break point, net point, and efficiency-related indicators, while the following indicators contributed most to the separation of two player categories: serve and return of serve points won (%), ace (%), peak serve speed, net points won (%), break point per return game, break point saved, winner and unforced error ratio, and dominance ratio. The research findings evidenced the decreased competitive balance in men’s competition during Grand Slams due to a rank-based seeding system, whereas coaches could use the information to fine-tune the training benchmarks and match planning.

## Introduction

The annual four major tennis tournaments (Australian Tennis Open, French Open, Wimbledon, and US Open), also known as Grand Slams, represent the highest level of professional tennis in the world (Gillet et al., 2009; Cui et al., 2017, 2019a). They not only own the longest tournament history, but brings together the top-ranked professional tennis players that compete along a period of 2 weeks, aspiring for the highest tournament prizes and points (Ma et al., 2013; Prieto-Bermejo and Gómez-Ruano, 2016; Reid et al., 2016; Cui et al., 2018). Moreover, the competitiveness of Grand Slams is also characterized by its best of five sets (best of three sets for female players) match format and an intensive draw of 128 players (Goossens et al., 2015; Prieto-Bermejo and Gómez-Ruano, 2016). Therefore, analyzing the form and function of match performance, these influential events for tennis players may provide a better understanding on how tennis tactics and strategies have developed in the elite level, and inform coaches of the technical, tactical, and physical demands of the most competitive situation. Consequently, knowledge gained from the quantification of performance could serve as a catalyst for optimized training and match arrangements (Reid et al., 2016; Woods et al., 2018, 2019).

In professional tennis matches, relevant literature has investigated the performance of players during different Grand Slams (O’Donoghue and Ingram, 2001; Ma et al., 2013; Tudor et al., 2014; Cui et al., 2018; Smith et al., 2018), focusing on topics such as match activity profiles (O’Donoghue, 2005; Johnson and McHugh, 2006), physiological responses of both male and female players (Reid and Duffield, 2014; Smith et al., 2018), effects of experience and individual features (Cui et al., 2017, 2019a), stroke and movement characteristics (Hizan et al., 2015; Reid et al., 2016; Whiteside and Reid, 2017), and evolution of certain performance aspect (such as serve speed, serve efficiency) (Cross and Pollard, 2009; Gillet et al., 2009). It has been well established that tennis match strategies and performance in these major events are conditioned by a myriad of factors such as court surfaces (O’Donoghue and Ingram, 2001; Cui et al., 2018), temperature and induced fatigue (Sell et al., 2013; Smith et al., 2018), gender difference (Hizan et al., 2015; Reid et al., 2016), player’s experience, relative quality, and anthropometric attributes (Vaverka and Cernosek, 2013; Cui et al., 2019a). Specifically concerning the opposition quality, it was reported that more experienced and taller players outperformed their peers in all of Grand Slams, especially achieving higher serve points won and break points conversion rate (Cui et al., 2018, 2019a). This extensive range of work provides insights into nuance of elite tennis match performance indicators and inform evidence-based training. Based on the extant findings, other aspects may also warrant consideration as research remains inclusive about how players’ match behavior is influenced by tournament seeding.

A seed in a tennis tournament is a player who is assigned with a preliminary ranking within the draw prior to the event. The process of seeding is usually determined by the tournament committee, using official Association of Tennis Professionals (ATP) rankings of players in the last 52 weeks as selection criterion (del Corral, 2009). Under such arrangement, seeded players will not need to play against each other until late in the tournament (the first and second seeds will not play against each other until the final), and they match with lower-ranked players in the first two rounds (Scheibehenne and Bröder, 2007). In Grand Slams, the seeding system of a 128-player main draw has evolved from initially including 8 seeded players to the current 32 seeds (del Corral, 2009). It is claimed that seeding system influences the competitive balance among all players, and as a result of audience preferences, it protects higher-rank contestants from early elimination (Du Bois and Heyndels, 2007; del Corral, 2009). Nonetheless, not all top 32 ranked players are necessarily seeded players as their previous performance on the events’ playing surfaces would also be considered (Scheibehenne and Bröder, 2007). Despite the fact that seeding could determine how much further players (especially low-ranked seeded players and non-seeded players) might proceed within Grand Slams, to date, research pertinent to the match performance of seeded and non-seeded players is scarce. The study by Whiteside et al. (2016) assessed players’ performance based on rankings and found that top-ranked players achieved more aces, serve points won, faster serve returns, and deeper ball placement than lower ranked players in the Australian Open. Of further relevance, del Corral (2009) reported a higher competitive balance in male players than female players during Grand Slams. However, there is a need to comprehensively evaluate the difference in match performance between seeds and non-seeds across all Grand Slam tournaments.

Since tennis performance at the highest level could be instructive to a fine-tuned training and match preparation process, it would be helpful to consolidate the understanding of how seeding system influences competitive match-play characteristics and to provide more realistic implications for players, especially those underdogs—non-seeded players. Therefore, the study was set to assess the difference in match performance indicators between seeded and non-seeded players in four Grand Slams men’s singles and establish the performance profiles of two groups of players, using key performance indicators that most differentiate them. It was expected that seeded players outperformed the non-seeded ones in serve-and-return, break point, net, and efficiency performance, while covering less distance (Cui et al., 2017).

## Materials and Methods

### Sample and Data

The study included 189 male professional players from 594 matches from four Grand Slams during the 2015–2017 season, which were 1,188 player observations (Australian Open: 148 individual players; French Open: 69 individual players; Wimbledon: 108 individual players; US Open: 108 individual players). The number of matches performed by individual players ranged from 1 to 17 (see Table 1). In the Australian Open (AO^1^), there were 269 seeded player observations (ranking: 13.2 ± 10.3) and 223 non-seeded player observations (ranking: 94.5 ± 80.8), French Open (FO^2^): 106 seeded player observations (ranking: 8.9 ± 7.1) and 48 non-seeded player observations (ranking: 96.3 ± 52.1), Wimbledon (W^3^): 162 seeded player observations (ranking: 12.8 ± 10.5) and 118 non-seed player observations (ranking: 97.8 ± 82.9), and US Open (US^4^): 157 seeded player observations (ranking: 12.6 ± 9.6) and 105 non-seeded player observations (ranking: 93.3 ± 91.5). The relevant match statistics of these players were obtained correspondent with tournament organizers. To avoid incompleteness of dataset, matches with withdrawn players were excluded and only those played on courts equipped with Hawk-eye tracking system (Hawk-Eye Innovations, Basingstoke, United Kingdom) were selected, which screened out 549 matches. The reliability of the match data and data collection process were previously tested and proved to be highly reliable (Cui et al., 2017). The study was undertaken under the approval of the local University Ethics Committee (Approval number: BSU2020009H) and all procedures were subject to all international standards and Declaration of Helsinki.

**TABLE 1:** Distribution and ranking (mean ± SD) of sampled players from four Grand Slams.

Grand Slam	Australian Open		French Open		Wimbledon		US Open	
Player Group	Non-seeded	Seeded	Non-seeded	Seeded	Non-seeded	Seeded	Non-seeded	Seeded
Number of individuals	124	42	46	24	81	34	75	38
World Ranking	94.5 ± 80.8	13.2 ± 10.3	96.3 ± 52.1	8.9 ± 7.1	97.8 ± 82.9	12.8 ± 10.5	93.3 ± 91.5	12.6 ± 9.6
Number of observations	223	269	48	106	118	162	105	157

### Performance Indicators

In this study, the seeded and non-seeded players were regarded as either independent or dependent variables in the following analysis. Match performance indicators related to players’ technical–tactical and physical behaviors in different Grand Slam tournaments were considered as dependent as well as independent variables. The raw match data were cleaned, organized, and computed to avoid misinterpretation of the player’s performance into 38 performance indicators after synthesizing from the previous literature (O’Donoghue, 2005; Gillet et al., 2009; Reid et al., 2016; Cui et al., 2018, 2019a). Table 2 shows each performance indicator according to the following categories: serving variables, returning variables, net point variables, break point variables, efficiency variables, and physical variables, and Supplementary Table 1 depicts their corresponding definitions.

**TABLE 2:** List of performance indicators.

Category	Indicator
Serving performance	Ace (%), Ace in deuce court (%), Ace in advantage court (%), serve winner (%), first serve in (%), first serve won (%), first serve won in deuce court (%), first serve won in advantage court (%), second serve (%), second serve won in deuce court (%), second serve won in advantage court (%), double fault (%), peak serve speed (km/h), first serve speed in deuce court (km/h), first serve speed in advantage court (km/h), second serve speed in deuce court (km/h), second serve speed in advantage court (km/h)
Returning performance	Return points won (%), return winner (%), return unforced error (%), first serve return won (%), second serve return won (%)
Net point performance	Net point won (%), net point won in total point won (%)
Break point performance	Break point per return game, break points won (%), break points saved (%)
Efficiency performance	Rally winner (%), rally forced error (%), rally unforced error (%), winner per unforced error ratio, dominance ratio (i.e., point won in return games/points lost in serve games)
Physical performance	Total distance covered in match (m), distance covered per set (m), distance covered per point (m)

### Statistical Analyses

Descriptive statistics (mean and standard deviation) were calculated for each performance indicator of seeded and non-seeded players, considering different tournaments. After testing the data normality distribution (Kolmogorov-Smirnov test), the independent *t* test was employed to compare the differences between seeded and non-seeded players in all indicators within distinct Grand Slam and while Mann–Whitney *U* test was run for other variables when the variables were not normally distributed. The meaningfulness of differences for *t* test was interpreted using standardized mean differences (Cohen’s *d*) as effect size statistics, which was calculated and interpreted by the following criteria: 0.2, trivial; 0.6, small; 1.2, moderate; 2.0, large; 4.0, very large; and ≥4.0, extreme large (Hopkins et al., 2009). For Mann–Whitney *U* test, *r* was used as effect size and interpreted according to the following thresholds: 0.3, small; 0.50, moderate; ≥0.5, large (Cohen, 1988).

**FIGURE 1:**
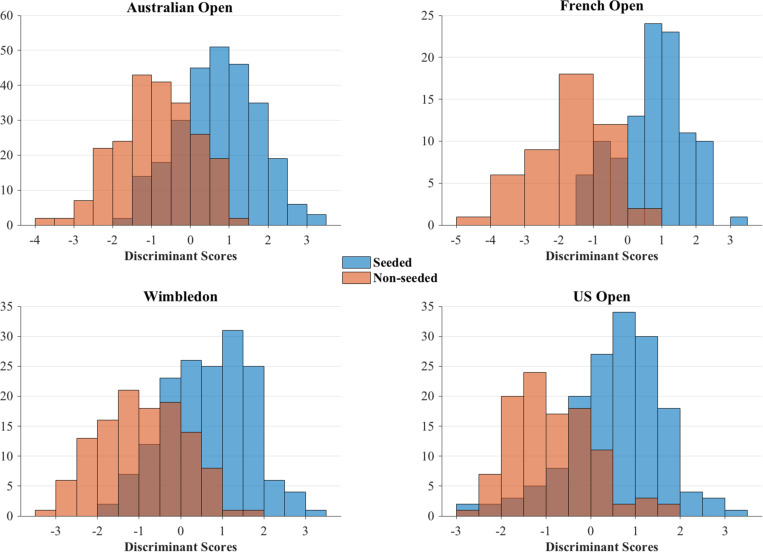
Distribution of discriminant scores for seeded and non-seeded player groups in the four Grand Slams.

**FIGURE 2:**
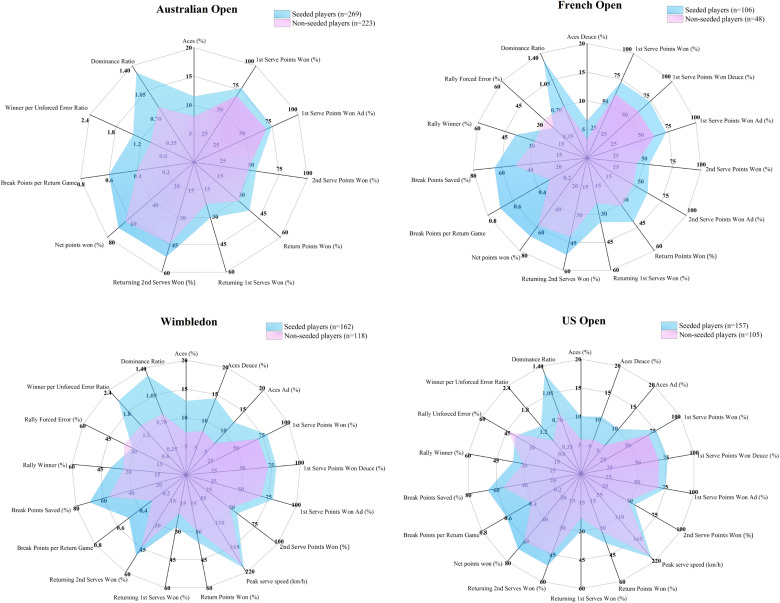
Performance profiles of two player groups using key indicators during the four Grand Slams. Each indicator was charted according to the corresponding scale.

Variables that were significantly different between two groups of players were then selected for the discriminant analysis, where seeded and non-seeded were taken as dependent variables. The analysis was to determine key performance indicators that best differentiate seeded and non-seeded players. Squared canonical correlation $\left(r_{\text{c}}^{2}\right)$ and partial eta square (η_p_^2^) were used as the effect sizes for discriminant functions (Field, 2013; Cui et al., 2019a). The interpretation of $r_{\text{c}}^{2}$ was as follows: 0.09, small; 0.25, moderate; large, ≥0.25, while the strength of η_p_^2^ was interpreted as follows: 0.06, small; 0.14, moderate; large, ≥0.14 (Cohen, 1988). In a significant discriminant function, performance indicators were considered a meaningful contributor to the differentiation of seeded and non-seeded players if their absolute value of the structural coefficient (SC) was greater than 0.30 (Sampaio et al., 2006). Afterward, the subsequent graphs of discriminant scores distribution were drawn using Matlab2018a (MathWorks, Inc., Natick, MA, United States) and the normative profiles of seeded and non-seeded players were plotted using means of key performance indicators in each Grand Slam (Figures 1, 2). The alpha level was set at *p* < 0.01 for all tests. All analyses were executed using Statistical Package for the Social Sciences 25 (SPSS Inc., Chicago, IL, United States).

## Results

Tables 3, 4 show the descriptive data and comparisons of the technical, tactical, and physical indicators between two player groups during the four major Slams. It was demonstrated that seeded players outperformed the non-seeded in serve and return, net point, break point, and efficiency-related indicators (*p* < 0.01, Cohen’s *d*: 0.25–1.45, *r*: 0.13–0.62). No significant difference was shown between two groups in Average 2nd Serve Speed AD, Return Winner, Return UE, Net Point Won in Total Points Won, and Total Distance Covered in Match.

**TABLE 3:** Descriptive statistics and comparisons of serve and return performance indicators for seeded and non-seeded players during Grand Slams.

Grand Slam	Australian Open			French Open			Wimbledon			US Open		
Player group	Non-seeded (*n* = 223)	Seeded (*n* = 269)	ES (90% CI for Cohen’s *d*)	Non-seeded (*n* = 48)	Seeded (*n* = 106)	ES (90% CI for Cohen’s *d*)	Non-seeded (*n* = 118)	Seeded (*n* = 162)	ES (90% CI for Cohen’s *d*)	Non-seeded (*n* = 105)	Seeded (*n* = 157)	ES (90% CI for Cohen’s *d*)
												
Indicators	Mean (SD)	Mean (SD)		Mean (SD)	Mean (SD)		Mean (SD)	Mean (SD)		Mean (SD)	Mean (SD)	
Aces (%)	8.0 (5.1)	11.5 (7.3)	0.49 (0.15)*	3.3 (2.5)	5.6 (3.7)	0.71 (0.27)*	7.4 (4.4)	12.9 (7.8)	0.87 (0.19)*	6.0 (4.0)	10.0 (6.5)	0.74 (0.2)*
Aces Deuce (%)	9.5 (6.8)	12.4 (8.4)	0.33 (0.15)*	3.3 (3.1)	6.5 (4.7)	0.8 (0.26)*	8.2 (5.9)	14.3 (9.2)	0.79 (0.19)*	6.3 (5.2)	10.6 (7.2)	0.67 (0.2)*
Aces Ad (%)	8.1 (6.2)	10.6 (8.2)	0.25 (0.15)*	3.8 (3.6)	5.1 (4.4)	0.33 (0.28)	7.4 (5.2)	12.4 (8.7)	0.69 (0.19)*	6.5 (4.6)	10.2 (7.7)	0.58 (0.2)*
Service Winners (%)	1.4 (1.2)	2.0 (1.6)	0.42 (0.17)*	0.7 (0.9)	0.8 (1.0)	0.07 (0.29)	1.1 (1)	1.4 (1.2)	0.28 (0.2)	1.6 (1.5)	2.5 (2.8)	0.4 (0.2)*
1st Serve In (%)	60.6 (6.5)	63.1 (6.4)	0.37 (0.15)*	60.3 (6.9)	62.7 (7.6)	0.33 (0.28)	61.7 (6.2)	64.0 (6.3)	0.36 (0.2)*	56.4 (7.0)	59.1 (6.6)	0.39 (0.21)*
1st Serve Points Won (%)	68.8 (8.0)	76.8 (8.4)	0.96 (0.15)*	61.1 (7.1)	72.5 (8.5)	1.45 (0.28)*	70.1 (8.5)	79.0 (8.1)	1.07 (0.2)*	68.2 (8.3)	75.9 (8.2)	0.92 (0.21)*
1st Serve Points Won Deuce (%)	71.8 (9.9)	76.1 (10.4)	0.4 (0.15)*	60.5 (7.8)	73.7 (10.7)	1.4 (0.27)*	71.8 (9.5)	79.2 (9.4)	0.78 (0.2)*	68.8 (11.9)	76.9 (9.2)	0.76 (0.21)*
1st Serve Points Won AD (%)	69.2 (11.3)	74.6 (10.7)	0.48 (0.15)*	61.7 (10.5)	72.5 (11.0)	1.0 (0.28)*	68.2 (11.1)	78.7 (9.9)	0.99 (0.2)*	68.7 (11.1)	74.7 (10.0)	0.57 (0.21)*
2nd Serve Points Won (%)	47.8 (9.4)	54.8 (10.8)	0.66 (0.15)*	43.8 (8.1)	54.5 (10.2)	1.16 (0.27)*	48.3 (10.1)	55.8 (9.8)	0.74 (0.2)*	47.7 (9.1)	54.0 (10.9)	0.63 (0.2)*
2nd Serve Points Won Deuce (%)	54.3 (13.2)	58.5 (14.4)	0.28 (0.15)*	49.6 (12.1)	59.2 (15.6)	0.69 (0.27)*	55.1 (15.4)	61.7 (14.4)	0.44 (0.2)*	54.5 (12.3)	59.9 (14.2)	0.4 (0.2)*
2nd Serve Points Won AD (%)	54.6 (14.5)	59.2 (14.2)	0.32 (0.15)*	46.7 (13.2)	60.9 (15.5)	0.98 (0.28)*	54.7 (13.7)	61.0 (15.7)	0.42 (0.2)*	55.6 (13.8)	61.3 (15.3)	0.39 (0.21)*
Double Faults (%)	9.7 (5.4)	9.0 (5.1)	0.04 (0.15)	8.9 (5.8)	7.2 (5.2)	0.32 (0.29)	10.1 (6)	8.2 (5)	0.34 (0.2)*	12.2 (5.7)	10.0 (6.1)	0.38 (0.21)*
Peak serve speed (km/h)	204.6 (11)	208.9 (11)	0.20^#^	199.7 (9)	205.2 (10)	0.27^#^	203.4 (9)	210.2 (10)	0.31^#^	204.4 (10)	209.2 (11)	0.21^#^
Average 1st Speed Deuce (km/h)	183.6 (10)	186.9 (10)	0.10	182.6 (9)	186.3 (9)	0.18	184.9 (9)	189.2 (9)	0.07	181.8 (10)	185.9 (10)	0.19^#^
Average 1st Speed AD (km/h)	184.1 (10)	187.8 (11)	0.07	180.9 (10)	181.7 (9)	0.02	185.3 (9)	192.1 (10)	0.05	180.9 (10)	185.2 (11)	0.16^#^
Average 2st Speed Deuce (km/h)	153.3 (10)	155.5 (11)	0.09	150.8 (10)	152.7 (9)	0.10	157.0 (11)	161.5 (12)	0.18	149.7 (10)	153.6 (11)	0.16^#^
Average 2st Speed AD (km/h)	149.2 (11)	150.9 (12)	0.08	144.5 (10)	145.9 (10)	0.11	155.3 (11)	159 (13)	0.07	146.0 (11)	149.4 (12)	0.10
Return Points Won (%)	31.0 (7.3)	38.5 (7.6)	0.95 (0.15)*	30.9 (6.7)	41.3 (8.1)	1.41 (0.28)*	29.4 (7.2)	36.1 (8.3)	0.85 (0.2)*	32.3 (7.2)	38.7 (8.1)	0.83 (0.21)*
Return Winners (%)	1.9 (1.8)	2.1 (1.7)	0.03 (0.16)	1.2 (1.3)	1.6 (1.6)	0.22 (0.28)	1.7 (1.5)	1.8 (1.6)	0.04 (0.2)	2.0 (1.7)	1.7 (1.7)	0.2 (0.21)
Return UE (%)	3.5 (2.9)	3.6 (2.5)	0.01 (0.16)	3.3 (2.4)	3.4 (2.0)	0.02 (0.3)	1.3 (1.5)	1.4 (1.5)	0.04 (0.2)	3.1 (2.5)	3.1 (2.7)	0 (0.21)
1st Serve Returns Won (%)	22.7 (8.1)	30.3 (8.5)	0.84 (0.15)*	24.1 (7.5)	34.2 (8.9)	1.21 (0.28)*	20.6 (8.2)	27.8 (9)	0.83 (0.2)*	23.3 (7.9)	29.8 (8.9)	0.78 (0.21)*
2nd Serve Returns Won (%)	44.4 (9.9)	51.6 (10.4)	0.66 (0.15)*	42.1 (9.2)	51.8 (10.1)	1.0 (0.28)*	44.4 (9.9)	49.5 (10.5)	0.5 (0.2)*	44.7 (10.5)	51.1 (9.9)	0.62 (0.21)*

* p < 0.01 for t test; ^#^p < 0.01 for Mann–Whitney U test. CI = confidence interval, confidence interval is shown only for Cohen’s d; Deuce = Deuce side of the service zone; AD = Advantage side of the service zone.

**TABLE 4:** Descriptive statistics and comparisons of net point, breakpoint, efficiency, and physical performance indicators for seeded and non-seeded players during Grand Slams.

Grand Slam	Australian Open			French Open			Wimbledon			US Open		
Player group	Non-seeded (*n* = 223)	Seeded (*n* = 269)	ES (90% CI for Cohen’s *d*)	Non-seeded (*n* = 48)	Seeded (*n* = 106)	ES (90% CI for Cohen’s *d*)	Non-seeded (*n* = 118)	Seeded (*n* = 162)	ES (90% CI for Cohen’s *d*)	Non-seeded (*n* = 105)	Seeded (*n* = 157)	ES (90% CI for Cohen’s *d*)
												
Indicators	Mean (SD)	Mean (SD)		Mean (SD)	Mean (SD)		Mean (SD)	Mean (SD)		Mean (SD)	Mean (SD)	
Net Points Won (%)	63.4 (12.0)	69.8 (12.4)	0.5 (0.15)*	58.2 (13.7)	67.4 (11.4)	0.72 (0.3)*	62.6 (11.6)	67.0 (12.0)	0.36 (0.2)*	62.0 (10.2)	68.4 (12.0)	0.58 (0.2)*
Net Points Won in Total Points Won (%)	15.8 (8.1)	14.8 (6.6)	0.11 (0.15)	15.5 (8.0)	14.8 (6.9)	0.09 (0.3)	17.5 (8.0)	17.7 (7.7)	0.03 (0.2)	15.9 (7.7)	16.2 (6.3)	0.05 (0.21)
Break Points per Return Game	0.4 (0.3)	0.6 (0.3)	0.40^#^	0.3 (0.2)	0.7 (0.3)	0.55^#^	0.3 (0.2)	0.5 (0.3)	0.33^#^	0.4 (0.2)	0.6 (0.3)	0.34^#^
Break Points Won (%)	34.7 (27.3)	40.8 (18.8)	0.16^#^	34.6 (24.0)	41.8 (18.2)	0.19	31.3 (27.9)	39.1 (20.5)	0.20^#^	34.9 (25.6)	40.6 (20.5)	0.13
Break Points Saved (%)	53.7 (18.4)	53.3 (30.8)	0.01	50.6 (15.9)	64.9 (20.7)	0.34^#^	56.1 (18.6)	70.0 (26.0)	0.33^#^	55.2 (17.4)	66.1 (25.0)	0.25^#^
Rally Winner (%)	28.8 (9.4)	33.1 (11.4)	0.19^#^	28.7 (7.7)	39.3 (9.4)	0.50^#^	32.9 (9.2)	39.7 (9.5)	0.35^#^	30.9 (9.5)	36.1 (9.0)	0.28^#^
Rally Forced Error (%)	32.7 (17.0)	32.4 (19.0)	0.05	29.4 (8.6)	23.6 (7.2)	0.31^#^	36.1 (9.2)	31.5 (7.6)	0.26^#^	25.3 (8.1)	24.2 (7.9)	0.06
Rally Unforced Error (%)	38.5 (15.3)	34.5 (14.3)	0.14^#^	42.0 (9.0)	37.0 (8.7)	0.24^#^	31.0 (8.8)	28.8 (9.3)	0.12	43.8 (9.9)	39.7 (9.6)	0.22^#^
Winner per Unforced Error Ratio	1.0 (0.6)	1.4 (0.7)	0.35^#^	0.8 (0.3)	1.4 (1.1)	0.48^#^	1.4 (0.8)	2.1 (1.1)	0.40^#^	0.9 (0.5)	1.3 (0.7)	0.41^#^
Dominance Ratio	0.8 (0.3)	1.3 (0.5)	0.56^#^	0.7 (0.2)	1.3 (0.6)	0.62^#^	0.8 (0.3)	1.3 (0.5)	0.56^#^	0.8 (0.3)	1.3 (0.5)	0.51^#^
Distance Covered in Match (m)	2233 (771)	2350 (916)	0.05	2203 (828)	2549 (862)	0.20	2002 (709)	2111 (706)	0.10	2222 (725)	2252 (844)	0.01
Distance Covered in Set (m)	597 (151)	650 (192)	0.13^#^	627 (168)	706 (175)	0.20	549 (140)	577 (142)	0.11	608 (152)	615 (164)	0.003
Distance Covered per Point (m)	9.8 (2.0)	10.5 (2.4)	0.15^#^	11.0 (2.5)	11.9 (2.6)	0.14	9.1 (2.2)	9.4 (2.2)	0.07	10.0 (2.2)	10.2 (2.3)	0.03

* p < 0.01 for t test; ^#^p < 0.01 for Mann–Whitney U test. CI = confidence interval, confidence interval is shown only for Cohen’s d.

Using performance indicators filtered from the previous analyses, the subsequent discriminant functions could effectively discriminate between the seed and non-seed players within each Grand Slam (*p* < 0.001, $r_{\text{c}}^{2}$: AO = 0.39, FO = 0.57, W = 0.39, and US = 0.33; η_p_^2^: AO = 0.15, FO = 0.25, W = 0.15, and US = 0.12, moderate to large effect sizes) and the reclassification rates are from 76.4 to 85.7%. Indicators that have meaningful contributions to the discriminant functions in distinct Slams are as follows: Ace, Ace Deuce, Ace AD, 1st Serve Points Won, 1st Serve Points Won Deuce, 1st Serve Points Won AD, 2nd Serve Points Won, 2nd Serve Points Won AD, Peak Serve Speed, Return Points Won, 1st Serve Returns Won, 2nd Serve Returns Won, Net Points Won, Break Points per Return Game, Break Points Saved, Rally Winner, Rally Forced Error, Rally Unforced Error, Winner per Unforced Error Ratio, and Dominance Ratio with |SC| s varying from 0.30 to 0.77 (see Table 5 for details on the results).

**TABLE 5:** Results of discriminant analysis and structure coefficients of input indicators for Grand Slams.

Grand Slam	Australian Open	French Open	Wimbledon	US Open
Indicators	Function SC	Function SC	Function SC	Function SC
Aces (%)	0.34*	0.27	0.52*	0.50*
Aces Deuce (%)	0.22	0.30*	0.48*	0.46*
Aces AD (%)	0.21	\	0.42*	0.39*
Service Winners (%)	0.23	\	\	0.27
1st Serve In (%)	0.22	\	0.17	0.24
1st Serve Points Won (%)	0.61*	0.57*	0.67*	0.65*
1st Serve Points Won Deuce (%)	0.25	0.54*	0.49*	0.47*
1st Serve Points Won AD (%)	0.3*	0.40*	0.62*	0.41*
2nd Serve Points Won (%)	0.43*	0.45*	0.46*	0.44*
2nd Serve Points Won Deuce (%)	0.19	0.27	0.28	0.28
2nd Serve Points Won AD (%)	0.20	0.39*	0.26	0.27
Double Faults (%)	\	\	−0.22	−0.27
Peak Serve Speed (km/h)	0.23	0.23	0.45*	0.32*
Average 1st Speed Deuce	\	\	\	0.26
Average 1st Speed AD	\	\	\	0.28
Average 2st Speed Deuce	\	\	0.27	0.22
Return Points Won (%)	0.62*	0.55*	0.53*	0.58*
1st Serve Return Won (%)	0.57*	0.48*	0.51*	0.54*
2nd Serve Return Won (%)	0.44*	0.40*	0.31*	0.44*
Net Points Won (%)	0.33*	0.30*	0.23	0.40*
Break Points per Return Game	0.53*	0.53*	0.44*	0.54*
Break Points Won (%)	0.17	\	0.2	\
Break Points Saved (%)	\	0.30*	0.37*	0.35*
Rally Winner (%)	0.25	0.49*	0.45*	0.40*
Rally Forced Error (%)	\	−0.31*	−0.35*	\
Rally Unforced Error (%)	−0.17	#	\	−0.3*
Winner per Unforced Error Ratio	0.36*	0.25	0.44*	0.47*
Dominance Ratio	0.78*	0.51*	0.75*	0.73*
Distance Covered in Set (m)	0.19	0.19	\	\
Distance Covered per Point (m)	0.21	\	\	\
Eigenvalue	0.642	1.325	0.637	0.487
Canonical Correlation	0.625	0.755	0.624	0.572
Wilks’ Lambda	0.609	0.43	0.611	0.673
Chi-square	237.055	119.787	131.381	98.19
Degree of freedom	24	20	23	25
Reclassification (%)	77.6	85.7	76.4	80.2

*SC = structure coefficient. “\” means that the variable was not used in discriminant analysis according to the previous analysis; # denotes the variable failing the tolerance test (minimum level = 0.001) and thus not used in the analysis; ^∗^|SC| ≥ 0.30.*

Figure 1 visualizes the distribution of the two groups of players in different Grand Slams using discriminant scores. The seeded players had higher discriminant scores in all competitions: Australian Open (seeded: 0.76 ± 1.04 vs. non-seeded: -0.92 ± 0.95), French Open (0.88 ± 0.95 vs. -1.95 ± 1.10), Wimbledon (0.88 ± 0.98 vs. -1.21 ± 1.03) and US Open (0.66 ± 1.03 vs. -0.98 ± 0.96). Finally, match performance profiles for both player groups are plotted using significant contributors to discriminant functions of different Grand Slams and are shown in Figure 2.

## Discussion

The current study analyzed the effect of player strength (being seeded and non-seeded) on tennis match performance in Grand Slams. The main focus was exploring key performance indicators that discriminate seeded and non-seeded players on different match locations. By doing so, it was possible to build the quantitative performance profiles of both player groups and account for opposition quality during training and match preparation process. Previous studies found that experienced, highly ranked, and taller players had better overall match performance than their peers during Grand Slams (Cui et al., 2017, 2019a), but failed to consider what differs between seeded and non-seeded players. It was stated by Neale (1964) in his Louis–Schmelling paradox that competitive balance has a great influence on individual sports such as tennis so that tournament organizers could alter it by giving advantages to stronger competitors. Therefore, quantifying difference margins in key performance indicator helps understand the competitive disadvantage of non-seeded players in such major tournaments (Klaassen and Magnus, 2001; del Corral, 2009). The results justified the assumption of better performance for seeded players in serve and return, break point, net point, and efficiency performance. Nonetheless, seeded players covered more distance than non-seeded players during match-play, which was contrary to the hypothesis. Finally, court surfaces had an influence on the extraction of key performance indicators differentiating two player groups.

Serve and return performance plays the utmost important role in tennis match tactics, and most of the points are finished in three to five rallies after serving and receiving the ball (O’Donoghue and Ingram, 2001; Mecheri et al., 2016; Cui et al., 2019b). Consistent with the previous literature, the current results revealed that serve and return performance indicators not only are key performance indicators to determine the match outcome but also discriminate different levels of players. In general, the seeded players were able to maintain around 75 and 55% in first and second serve points won (%) and 30 and 50% in first and second serve returns won (%) during four Grand Slams, which outperformed the non-seeded players by 10%. When looking into the other serve-relevant aspects, it was shown that in AO, W, and US, seeded players could achieve nearly one ace out of 10 serves. While this statistic declined to around 6% in FO, it was still 3–4% higher than the non-seeded counterparts. In addition, it is noteworthy that the peak serve speed of seeded players was higher than non-seeded ones in all events. Finally, in terms of ball-returning, the seeded players also obtained more return points, especially during the second returns, where they could win around 50% of points. On the one hand, it is evident that the seeds have better familiarity with different court surfaces and adjust their techniques accordingly to maximize their advantage at the start of points. On the other hand, this proves that they possess more advanced psychological and technical attributes, which allow them to eliminate the opponent’s advantage at serve (Gillet et al., 2009; Cui et al., 2017).

Usually, the net point consists of three forms of point-ending, namely, volleying, smashing, and net-approaching. Although both seeded and non-seeded players had similar percentage of Net Points Won in Total Points Won during the four Grand Slams, the former showed better net point efficiency (winning over 67% of all net points) than the latter (58–63%). This reveals that seeded players could create and seize the timing of the net point and possess better net point techniques than ordinary professional players, which coincides with the results of a former study in that higher-ranked and experienced tennis players were more technically well-developed than their peers (Cui et al., 2017). Future research should inspect specific tactical scenarios of their net performance, such as net-approaching after rallies, serve and volley, return and volley, and being forced to approach the net, so as to provide in-depth feedback to representative training design.

In terms of the break point performance, the results suggest that the seeded players not only could get more break point opportunities in the opponent’s serve and achieve higher winning percentage, but also saved more break points than the non-seeded players. Moreover, the discriminant analysis underpins the importance of break point opportunities as discriminator of two player groups. It is evident that seeded players displayed better return performance in opponents’ service games, as well as more stable psychological status that endowed themselves with advantage in such critical points (O’Donoghue, 2012). Future study should further consider these performances in relation to the point outcome to unveil what behaviors seeded players demonstrate to succeed in this situation. Finally, it would be beneficial that more varied break point scenarios be simulated in training, forcing players to adjust their tactical solutions and to be mentally prepared (Meffert et al., 2019).

Previous findings highlighted that winner- and unforced error-related indicators are important in assessing high-level tennis players’ performance (Filipcic et al., 2015; Cui et al., 2017). Moreover, it was suggested that underdogs in major sports events (non-seeded players in this case) tend to use riskier strategies than favorites (seeded players) (del Corral, 2009). However, the current results showed that the seeded players achieved relatively higher number of winners during both whole match and rally, but also maintained lower unforced and forced errors than their non-seeded counterparts. It is evident that a rank-based seeding system decreases competitive balance in men’s game during Grand Slams (del Corral, 2009), and with technical–tactical superiority and mental tenacity during match-play (Cowden, 2016; Reid et al., 2016), seeded players are favored to play with more effective risky strategies.

Although an empirical comparison among the four major Slams reveals that players covered more running distances on French Open clay courts and less on Wimbledon grass courts, there is no significant difference between the seeded and non-seeded players. As knowing the average running distance of 2,000–2,500 m and 9.1–11.9 m within the entire match and a single point for all players would not necessarily advance our understanding of how two competing players move during ball-interchange, future studies should shed light on point-level running patterns such as change of directions, acceleration, and deceleration through ball/player tracking, so as to gain insights into difference of player levels in altering hitting direction, speed, and rhythm. As a practical application, our results could still set a benchmark for tennis training, where coaches are suggested to focus on building the offensive playing patterns of players under no more than five rallies with the intention to win each serving and receiving point as soon as possible. Furthermore, fitness-specific physical training (that centers on the development of player’s aerobic and anaerobic capacity and speed) and game-specific physical training (related to ball-striking and footwork drills) should be refined to include more high-intensity, short-interval bouts so as to induce better muscle and mental recovery at a point-to-point level.

Notwithstanding novel findings provided by the study, this study failed to further look into the influence of seeding system on match outcome, for example, analyzing players’ performance during different confrontations: seeded vs. seeded, seeded vs. non-seeded, and non-seeded vs. non-seeded. Meanwhile, including recent performance, e.g., represented by ELO rating (Kovalchik, 2020), rather than players’ rankings might offer more constant information about their competitive match-play. Finally, the current findings need to be verified in the female counterparts, as the match format of women’s game is comparatively shorter than the men’s (i.e., best of three sets) and the match pattern is more characterized by baseline performance (Cui et al., 2018).

## Conclusion

Within the four major Grand Slam tournaments’ men’s singles matches, the seeded players outperformed non-seeded players in serve and return, net points, break points, and game efficiency-related indicators. Serve and return of serve points won (%), ace (%), peak serve speed, first serve speed, net points won (%), break point per return game, break point saved, winner and unforced error ratio, and dominance ratio turned out to be most meaningful discriminators of the two player groups and can be used as valid player assessment indices during practices.

## Data Availability Statement

The datasets generated for this study are available on request to the corresponding author.

## Ethics Statement

The studies involving human participants were reviewed and approved by Research commission of Beijing Sport University. Written informed consent for participation was not required for this study in accordance with the national legislation and the institutional requirements.

## Author Contributions

YC, M-ÁG, and YZ: conceptualization and data curation. YC: formal analysis. YC, HL, YZ, and RW: investigation. YC and M-ÁG: methodology, software, writing – original draft preparation, and designing the experiments and performing the statistical analysis. YC and YZ: visualization. YC, RW, M-ÁG, HL, and YL: writing – review and editing. HL and YZ: funding acquisition. YC, YZ, HL, and RW: writing and revising the manuscript. M-ÁG, HL, and YL: supervising the design and reviewing the manuscript. All authors have made a substantial and direct contribution to manuscript, and approved the final version of the manuscript.

## Conflict of Interest

The authors declare that the research was conducted in the absence of any commercial or financial relationships that could be construed as a potential conflict of interest. The reviewer BG declared a past co-authorship with one of the authors M-ÁG.

## Footnotes

1 www.ausopen.com

2 www.rolandgarros.com

3 www.wimbledon.com

4 www.usopen.org
