# Supplementary material for: Effect of a Seeding System on Competitive Performance of Elite Players During Major Tennis Tournaments
Source: Front Psychol. 2020 Jun 26;11:1294. doi: 10.3389/fpsyg.2020.01294 (PMC7332749; doi:10.3389/fpsyg.2020.01294)
Supplement: Supplementary file 1 [file Table_1.DOCX]

| Supplementary Table 1. Definitions of tennis performance indicators used in the current study | |
| --- | --- |
| Indicator | **Definition** |
| Aces (%) | % of aces in total number of serves (ace is a successful serve that opponent fails to touch by her racquet) |
| Aces in deuce court (%) | % of aces in serves directed to deuce court side |
| Aces in advantage court (%) | % of aces in serves directed to advantage court side |
| Service winners (%) | % of service winners in total services (serve winner is a serve that is touched by opponent with her racquet but not returned into the court) |
| First serve in (%) | % of first serves that were in play |
| First serve points won (%) | % of points won when first serves were in play |
| First serve points won in deuce court (%) | % of points won when first serves were served into the deuce side |
| First serve points won in advantage court (%) | % of points won when first serves were served into the advantage side |
| Second serve points won | % of points won when second serves were in play |
| Second serve points won in deuce court (%) | % of points won when second serves were served into the deuce side |
| Second serve points won in advantage court (%) | % of points won when second serves were served into the advantage side |
| Double Faults (%) | % of double faults in total number of second serves |
| Peak serve speed (km/h) | Maximum serve speed for the match |
| First serve speed in deuce court (km/h) | Average first serve speed for the match when serving into deuce court |
| First serve speed in advantage court (km/h) | Average first serve speed for the match when serving into advantage court |
| Second serve speed in deuce court (km/h) | Average second serve speed for the match when serving into deuce court |
| Second serve speed in advantage court (km/h) | Average second serve speed for the match when serving into advantage court |
| Return points won (%) | % of points won when returning serves |
| Return winners (%) | % of return winners in total serve returns |
| Return unforced errors (%) | % of unforced errors in total serve returns |
| First serve return won (%) | % of points won when returning first serves |
| Second serve return won (%) | % of points won when returning second serves |
| Net points won (%) | % of points won when attacking the net |
| Net points won in total points won (%) | % of net points won in total points won during the match |
| Break points per return game | Number of break points had in every opponent’s service game |
| Break points won (%) | % of break points converted in opponent’s service game |
| Break points saved (%) | % of break points served in own service game |
| Winner in rally (%) | % of winners in points won during baseline rally |
| Forced error in rally (%) | % of forced errors in points lost during baseline rally |
| Unforced error in rally (%) | % of unforced errors in points lost during baseline rally |
| Winner per unforced error ratio | Number of winners in every unforced error |
| Dominance ratio | Calculated as: % of points won in opponent’s serves / % of points lost in own serve |
| Total distance covered in match (m) | Mean distance covered in a match by individual player |
| Distance covered per set (m) | Mean distance covered in each set by individual player |
| Distance covered per point (m) | Mean distance covered in each point by individual player |
